# NOX-Dependent Signaling Dysregulation in Severe COVID-19: Clues to Effective Treatments

**DOI:** 10.3389/fcimb.2020.608435

**Published:** 2020-12-15

**Authors:** Simona Damiano, Concetta Sozio, Giuliana La Rosa, Mariarosaria Santillo

**Affiliations:** Dipartimento di Medicina Clinica e Chirurgia, Università di Napoli “Federico II”, Napoli, Italy

**Keywords:** coronavirus disease 2019, severe acute respiratory syndrome coronavirus 2, NOX enzymes, angiotensin converting enzyme 2, hypertension, cardiovascular diseases, diabetes, obesity

## Introduction

The ongoing pandemic disease named by the World Health Organization (WHO) coronavirus disease 2019 (COVID-19), is caused by Severe Acute Respiratory Syndrome Coronavirus 2 (SARS-CoV-2), the seventh confirmed RNA single strand beta coronavirus infecting the human beings. According to the WHO, to date (November, 10^th^ 2020), SARS-CoV-2 has affected 50,459,886 people and caused 1,257,523 deaths in the world.

Around 80% of people affected by SARS-CoV-2 infection is asymptomatic or present mild to moderate symptoms, mostly restricted to the upper and conducting airways, often including anosmia and ageusia. The other 20% develops a more critical clinical situation needing hospitalization, which progress, in 5% of cases, in a severe respiratory syndrome requiring ventilatory support and even death (Domingo et al., 2020).

SARS-CoV-2 infects predominantly endothelial cells which express Angiotensin Converting Enzyme (ACE) 2 plasma membrane protein to which the spike protein of SARS-CoV-2 binds (Wan et al., 2020). Therefore, ACE2 represents the receptor of SARS-CoV-2 and ACE2 deficiency related to viral destruction of ACE2 expressing cells, causes severe acute respiratory syndrome and other symptoms related to endothelial cells infection (See *NOX2 Activation in COVID-19*). However, endothelial cells are not the only target cells of SARS-CoV; ACE2 is also expressed in respiratory epithelium, in type II pneumocytes, and in cells of cardiovascular, gastrointestinal, renal, and central nervous systems as well as adipose tissue (Hamming et al., 2004). COVID-19 is therefore a multi-organ disease as the effects of viral infection are extended to a wide range of tissues and organs and, in many cases, death occurs from non-respiratory causes such as renal failure or ischemic episodes (Zaim et al., 2020).

Reactive oxygen species (ROS) derived from NADPH oxidase enzymes (NOXs) and oxidative stress are involved in viral pathogenesis (Khomich et al., 2018). NOX enzymes, present in cell and phagosome membranes, belong to a family of six members (NOX1-5 and DUOX1 and 2) which catalyze the dismutation of superoxide anion to hydrogen peroxide and water (Bedard and Krause, 2007). NOX-driven ROS are signaling molecules regulating numerous physiological functions (Damiano et al., 2015; Damiano et al., 2019). However, a growing body of evidence points to a connection between exceeding NOX-derived ROS and multiple chronic diseases including hypertension, diabetes, and cardiovascular disease as well as obesity (Sahoo et al., 2016).

Among patients developing the severe form COVID-19, a high percentage suffer of pre-existing pathological conditions like hypertension (57%), diabetes (34%), obesity (42%), and cardiovascular diseases (20–40%) which are also main contributing factors for COVID-19-related deaths (Zhou et al., 2020).

Highly effective therapeutics to prevent or treat the severe form of COVID-19 infection are still lacking. We believe that understanding the common denominator of COVID-19 co-existing pathological conditions and why they worsen the outcome of COVID-19 is a current need for the development of more effective interventions or even to take prevention measures to protect subjects at higher risk.

A possible common pathogenic mechanism of the main comorbidities associated with the development of the severe form of COVID-19 is the activation of the ROS generating NADPH oxidase enzymes (NOXs) and the associated oxidative stress (See *NOX Activation Is a Common Hallmark of Main Comorbidities Associated to Severe COVID-19*). Of note, endosomal NOX activation is essential for SARS-CoV cell infection and some studies have demonstrated the induction of a serum marker of NOX activation in patients affected by COVID-19 (Violi et al., 2020).

Taking into account all these evidences, we propose that a pre-existing NOX pathway dysregulation could be a determining factor in the development of the severe form of COVID-19 infection and in the onset of complications worsening the clinical outcome of disease.

## NOXs and Viral Diseases

### NOXs in SARS-CoV Infection

Oxidative stress is a hallmark of severe SARS-CoV infections including SARS-CoV-2 (Cecchini and Cecchini, 2020). Before the onset of COVID-19 pandemic, it has been shown that NOX2 is activated in the endosomal compartment by different types of RNA and DNA viruses employing endosomes as their primary entry mechanism (To et al., 2017). It has been proposed that the interaction between viral RNA/DNA and endosomal toll-like receptor 7 (TLR7) activates this latter which, in turn, induces NOX2, increasing ROS (To et al., 2017; DiNicolantonio and McCarty, 2020). Even if it is still unknown, it is reasonable to hypothesize that SARS-CoV-2, as well as others single stranded RNA and DNA viruses entering cells *via* endosomes, relies on the same TLR7/NOX2-dependent mechanism to infect cells and to circumvent antiviral responses inducing oxidative stress and cell injury. In viral infections, NOX derived ROS, contribute to inflammation and tissue damage (Khomich et al., 2018). However, additional functions have been attributed to NOXs in viral establishment and on the onset of complications. It has been shown that NOX enzymes play a direct role in the early events of retrovirus life cycle (Kim and Wong, 2013), while, in rhinovirus infections, they determine an impairment of epithelial barrier functions, thus facilitating bacterial transmigration (Comstock et al., 2011).

In general, as compensatory mechanism, oxidative stress induces an antioxidant response through nuclear factor E2-related factor 2 (Nrf2) pathway activation. At low levels of ROS, Nrf2 is associated in the cytoplasm with kelch-like ECH-associated protein 1 (Keap1), which targets Nrf2 to proteasomal degradation. When ROS are generated at high levels, Nrf2 dissociates from Keap1, translocates into the nucleus, and binds the antioxidant response element (ARE) of the promoters of target genes encoding mainly antioxidant enzymes like superoxide dismutases, catalase, peroxiredoxins, and glutathione peroxidases (Khomich et al., 2018).

**Figure 1:**
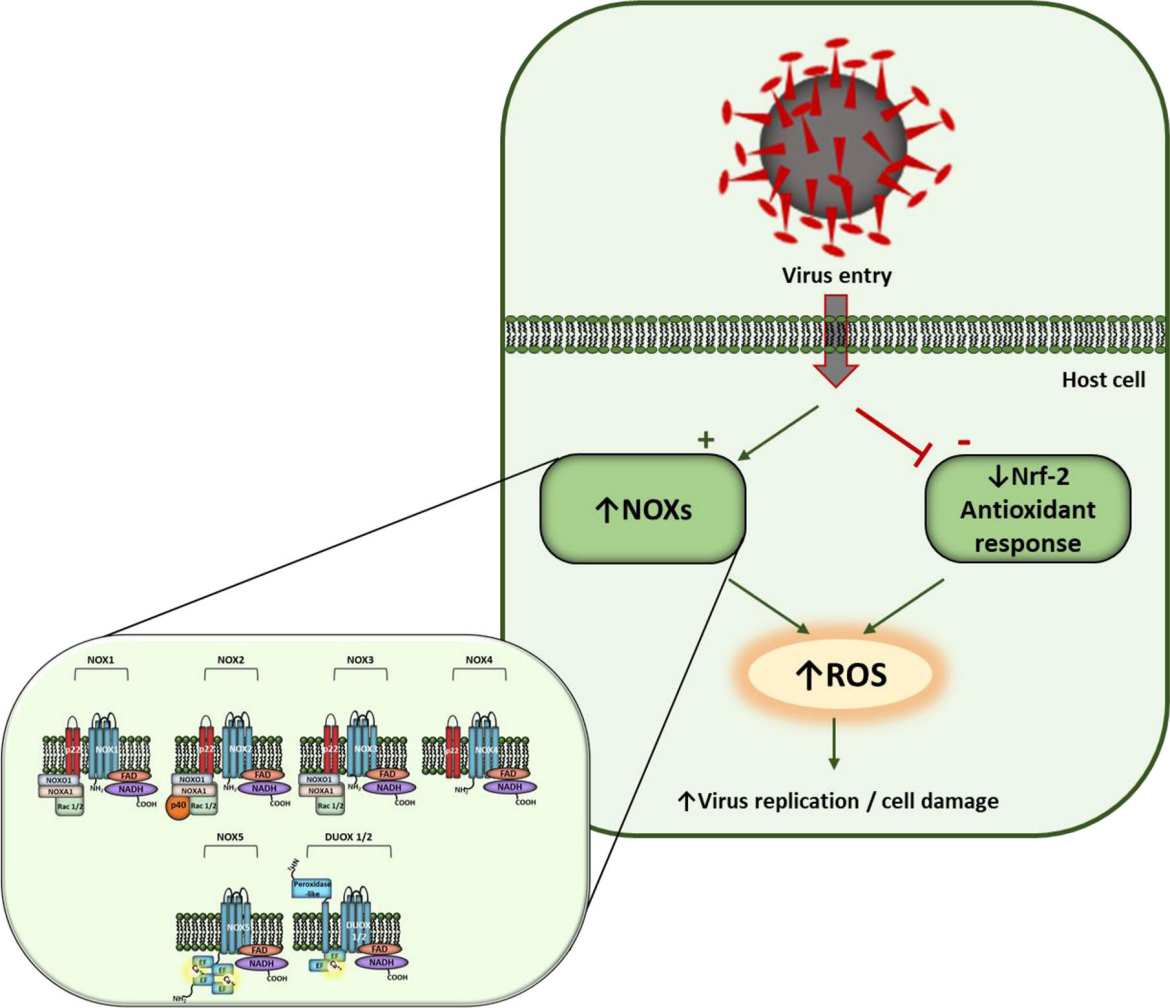
Hypothesis of redox control of host cells in SARS-CoV-2 infection. Virus entry activates NOXs and inhibits Nrf-2 antioxidant response inducing ROS levels. NOX family members and their regulatory subunits are shown in detail on the left. NOX1-3 comprise two membrane subunits (NOX1-3 and p22phox), that represent the catalytic core of the enzyme, and different cytosolic subunits which translocate to membrane upon activation. NOX4 is constitutively active when associated with p22phox subunit. Nox5 and Duox1/2 activation involves Ca^2+^ binding to EF-hand domains.

In viral infections, oxidative cell damage can be associated to an inhibition of Nrf2 pathway (Komaravelli and Casola, 2014). Noteworthy, it has been shown that the expression of Nrf-2 related antioxidant gene expression is suppressed in biopsies obtained from COVID-19 patients (Olagnier et al., 2020) (**Figure 1**). On this basis, it has been hypothesized that antioxidant therapy, endosomal-targeted ROS inhibitors (To et al., 2017), or Nrf-2 activators could suppress pathogenicity of many viruses, including SARS-CoV-2 (Cuadrado et al., 2020).

### NOX and ACE2/Ang(1-7)/Mas Receptor Signaling

About the pathogenetic mechanisms of COVID-19 and its complications, the downregulation of ACE2/Ang (1-7)/Mas axis exerts a pivotal role. ACE2 is a master regulator of the renin-angiotensin system (RAS) mainly by converting Ang (angiotensin) I and Ang II into Ang (1–9) and Ang (1–7), respectively (Wang et al., 2020). Ang (1–7), through the interaction with Mas receptor, negatively regulates AngII/AT1, thus contrasting the vasoconstrictive, proinflammatory, and prothrombotic effects of this pathway. ACE2/SARS-CoV-2 interaction results in a loss of ACE2 which is driven into the cells by endocytosis and cleaved by proteolysis. Therefore, the loss of ACE2 has two negative effects. The first is an upstream accumulation of Ang II and hyperactivation of the AngII/AT1R pathway; the second one is the downregulation of the counterregulatory Ang (1-7)/Mas receptor pathway which antagonizes AngII/AT1R. The downregulation of ACE2 ultimately can explain the severe multi-organ symptomatology subsequent to SARS-CoV-2 infection.

Interestingly, experimental data link NOXs and ACE2/Ang (1–7)/Mas receptor axis. In transgenic mouse overexpressing ACE2 in neurons, the hyperactivation of ACE2/Ang (1–7)/Mas axis has a protective effect against ischemic injury by preventing ischemia-induced NOX/ROS signaling pathway activation, especially in older animals (Zheng et al., 2014). Therefore, as opposed to ACE/AngII axis which activates NOXs, it seems that ACE2/Ang (1–7)/Mas axis activation is associated with NOXs inhibition.

### NOX2 Activation in COVID-19

It has been hypothesized that oxidative stress could play a prominent role in determining cardiovascular complications in COVID-19 and that oxidative stress is linked to NOX activation (Loffredo and Violi, 2020).

Relevant experimental data are now emerging about the relationship between NOXs-dependent pathway and the course of COVID-19. NOX2 activation is associated with severe disease and thrombotic events in COVID-19 patients (Violi et al., 2020). The soluble Nox2-derived peptide (sNox2-dp), a serum marker of NOX2 activation, has been measured in 182 patients hospitalized for COVID-19. The data evidenced that sNox2-dp values were higher in COVID-19 patients compared to controls and in severe *versus* non-severe COVID-19 affected subjects.

## NOX Activation Is a Common Hallmark of Main Comorbidities Associated to Severe COVID-19

The main COVID-19 comorbidity includes hypertension, cardiovascular diseases, obesity, and diabetes, all of which are associated to NOX signaling dysregulation. Hypertension is an important risk factor for other cardiovascular diseases such as coronary artery disease, stroke, heart failure, peripheral vascular disease, chronic kidney disease, and atherosclerosis, as well as for the development of severe COVID-19. NOXs are the main source of ROS within the vascular system. Many data have shown that hypertension-associated vascular functional and structural changes are attributable to NOX-driven intravascular ROS generation. Indeed, Ang II, a well-known pro-hypertensive factor, augments blood pressure activating NOX *via* AT1 receptor (Okabe et al., 2020). Among NOX isoforms, NOX5 exerts a relevant role in blood pressure control. In support of these findings, in a recent genome-wide association study (GWAS) of 475,000 subjects, NOX5 was recognized as an important blood pressure associated gene (Kraja et al., 2017).

Diabetes mellitus, as well, has been identified as an important risk factor for severe COVID-19. A meta-analysis including 33 studies have shown that diabetes is associated with a two-fold increase in mortality and severity of COVID-19, as compared to non-diabetics (Kumar et al., 2020). Studies in type 2 diabetes (TD2) models revealed that anomalous NOX activation contributes to endothelial dysfunction. In TD2 patients hyperglycemia is coupled with inflammation and oxidative stress leading to endothelial dysfunction (Park et al., 2009). Among the endothelial cellular events triggered by hyperglycemia, NOXs are key players involved in oxidative stress generation (Feduska and Tse, 2018).

Obesity represents a high risk factor for the development of severe COVID-19 infection (Sattar et al., 2020). Experimental data mechanistically link the development of obesity with NOX activation. Obesity, along with obesity-associated conditions such as metabolic syndrome and diabetes, is associated with increased NOX-driven ROS production in key organs such as adipose tissue, skeletal muscle and vasculature. The resulting oxidative stress impairs numerous physiological processes like insulin signaling, endothelial functions, and others which overall lead to inflammation and development of pathological conditions (DeVallance et al., 2019). Is has been proposed that ROS derived by NOX4, the major adipocyte isoform of NADPH oxidase, may play a role in the onset of insulin resistance and adipose tissue inflammation during the development of obesity in mice fed a high-fat, high-sucrose diet (Den Hartigh et al., 2017).

In general, NOX pathway-dependent dysregulation is a key determinant in the loss of endothelial functions (Meza et al., 2019) which is a common feature also of other co-existing pathologies associated to COVID-19 worsening its outcome. Moreover, in the attempt to explain the common thromboembolic complications of COVID-19, it has been proposed that SARS-CoV-2 infection of endothelial cells induces luminal expression of tissue factor (TF) which activate extrinsic clotting (DiNicolantonio and McCarty, 2020). In the proteolytic cascade triggered by TF leading to blood coagulation the activation of NADPH oxidase is mandatory (Herkert et al., 2004).

## Discussion

The elevated number of death associated to COVID-19 arises from the inadequacy of current treatments unable to combat SARS-CoV-2 infection or to prevent severe complications and ultimately death. Therefore, exploring new treatment options interfering with signaling pathways involved in the pathogenesis of COVID-19 is an urgent need.

The data discussed in the present opinion paper suggest that reducing oxidative stress could improve the poor outcome that characterized severe COVID-19. Due to the central role exerted by NOXs pathway dysregulation and related oxidative stress in the main comorbidity associated to severe COVID-19, and considering its involvement in SARS-CoV viral infection mechanism, targeting NOX enzymes seems a promising strategy to treat COVID-19 and to prevent severe complications.

There are currently no drugs available to selectively block NOXs enzymes approved for their use in human subjects. Experimentally, it has been shown that NOX inhibitors such as diphenyleneiodonium (DPI), apocynin and 4-(2-aminoethyl) benzenesulfonyl fluoride (AEBSF), or peptidic inhibitors are effective in mitigating symptomatology in different animal models of disease (Altenhöfer et al., 2015). However, the reduction of oxidative stress by many substances used in humans is associated with downregulation of NOX signaling. Indirect evidence of the efficacy of treatments for COVID-19 influencing NOXs are represented, among others, by metformin. Contrasting data are present in literature about the association between metformin and decreased mortality in patients with COVID-19 and diabetes. However, a meta-analysis including five studies with a total of 6,937 patients showed that metformin use is associated with reduction in mortality rate from COVID-19. Similar findings are reported in a retrospective study by Luo et al. (2020). Moreover, in obese or T2DM subjects affected by COVID-19, metformin was associated with decreased mortality in women, while, among men, no significant reduction in mortality was observed (Bramante et al., 2020). This sex specific effect of metformin in protecting by COVID-19 was explained with the finding that metformin reduces the pro-inflammatory cytokine TNFalfa in female but not in male. Different hypotheses have been formulated to explain metformin molecular mechanism in protecting from severe form of COVID-19. However, importantly, metformin seems to inhibit the inflammatory response and to modulate NOX (Najafi et al., 2018).

In addition, natural compounds like polyphenols, could have a potential preventive role against the onset of severe COVID-19. These natural compounds have antioxidant properties; however, like some enzymatic antioxidants (Damiano et al., 2013; Damiano et al., 2020), their biological effects are related to the ability to modulate cell-signaling pathways rather than to their antioxidant activity (Damiano et al., 2018). Of note, polyphenols, which have been proposed to serve for prevention and therapy of COVID-19 for their anti-inflammatory properties, have an inhibitory effect on NOXs activity (Yousefian et al., 2019). The list of substances that could improve oxidative stress through inhibition of NOX-dependent pathways is broad and those reported here represent only some intriguing examples.

In conclusion, we believe that the proposed unified point of view of the clinical picture associated with the development of severe COVID-19 highlighting the role of NOXs as key molecular determinants, may provide important clues for the protection of subjects at higher risk and for the development of new targeted therapies fighting the severe forms of illness.

## Author Contributions

MS and SD conceived and contributed to the writing of the manuscript. CS and GLR contributed to the writing of the manuscript. All authors contributed to the article and approved the submitted version.

## Conflict of Interest

The authors declare that the research was conducted in the absence of any commercial or financial relationships that could be construed as a potential conflict of interest.
